# Mucosal healing of small intestinal stricture is associated with improved prognosis post-dilation in Crohn’s disease

**DOI:** 10.1186/s12876-022-02300-2

**Published:** 2022-05-04

**Authors:** Shuji Hibiya, Kazuo Ohtsuka, Kento Takenaka, Ami Kawamoto, Yusuke Matsuyama, Yumi Udagawa, Maiko Motobayashi, Hiromichi Shimizu, Toshimitsu Fujii, Eiko Saito, Masakazu Nagahori, Ryuichi Okamoto, Mamoru Watanabe

**Affiliations:** 1 Department of Gastroenterology and Hepatology, Graduate School, Tokyo Medical and Dental University, 1-5-45 Yushima, Bunkyo-ku, Tokyo, 113-8519 Japan; 2 Endoscopic Unit, Tokyo Medical and Dental University Hospital, Tokyo, Japan; 3 Department of Global Health Promotion, Tokyo Medical and Dental University, Tokyo, Japan; 4 TMDU Advanced Research Institute, Tokyo Medical and Dental University, Tokyo, Japan

**Keywords:** Crohn’s disease, Small intestinal stricture, Endoscopic balloon dilation

## Abstract

**Background:**

Small intestinal stricture is a major cause for surgery in Crohn’s disease (CD). Endoscopic balloon dilation (EBD) is performed for small intestinal strictures to avoid surgery, often repeatedly. However, factors that are associated with prognosis after EBD of small intestinal strictures remain poorly investigated. Mucosal healing is the therapeutic target in CD. We aimed to investigate the impact of mucosal healing defined by the presence of ulcers at the small intestinal stricture site on the prognosis of EBD in CD patients.

**Methods:**

We retrospectively included patients with CD who underwent initial EBD for endoscopically impassable small intestinal strictures from January 2012 to March 2020 at a single center. The association between presence of ulcer at the stricture site and surgery after EBD was examined by Cox proportional hazards model.

**Results:**

Of the 98 patients included, 63 (64.3%) had ulcer at the stricture site. 20 (31.7%) of these patients underwent surgery for the stricture in due course, whereas 4 (11.4%) of the patients without ulcer of the stricture underwent surgery. In multivariate analysis, patients with ulcer of the stricture had a significantly higher risk for surgery than those without ulcer (hazard ratio 4.84; 95% confidence interval 1.58–14.79).

**Conclusion:**

Mucosal healing at the stricture site indicated a favorable prognosis after EBD for small intestinal strictures in CD.

**Supplementary Information:**

The online version contains supplementary material available at 10.1186/s12876-022-02300-2.

## Background

Crohn’s disease (CD) is a chronic condition characterized by inflammation in all layers of the digestive tract [1]. Repeated inflammation leads to intestinal stricture formation, which, in severe cases, requires intestinal resection. The development of balloon-assisted enteroscopy (BAE) [2] has made endoscopic balloon dilation (EBD) of small intestinal strictures possible, consequently avoiding or delaying surgery in some cases [3, 4]. Performing EBD on small intestinal strictures has been reported to be effective [5–12]. However, some patients do not improve or require re-dilation in a short period.

Although the association between the technical conditions of dilation and post-dilation prognosis has been reported [11–13], the association between disease activity and post-dilation prognosis remains unclear. A meta-analysis of 33 studies published between 1991 and 2013 reported that the presence of inflammation at the stricture site is not associated with long-term prognosis after EBD [14], while a multicenter report of 273 patients found that the presence of activity tended to increase the likelihood of requiring re-dilation or surgery [15]. However, previously reported analyses were based on data using colonoscopy, and reports on small intestinal lesions remain limited. The ECCO technical review [16] explains that the absence of ulcer is associated with a good prognosis after EBD, but most of the reported analyses [17] are for anastomotic strictures, with only two cases of CD-induced stricture. Thus, the relationship between disease activity and prognosis after EBD for small intestinal strictures remains unclear.

If mucosal healing is achieved, progression to intestinal damage can be prevented, thereby improving the prognosis [18, 19]. In a recent report, patients who achieved endoscopic mucosal healing of small intestinal lesions had an improved prognosis, but the effect of endoscopic dilation for strictures was not considered [20]. We hypothesized that patients with mucosal healing defined by the absence of ulcers at the stricture site, would have improved prognosis after EBD for CD strictures.

Thus, this study aimed to investigate the association between the presence of ulcer of small intestinal stricture and prognosis after EBD in patients with CD.

## Methods

### Patients

**Fig. 1:**
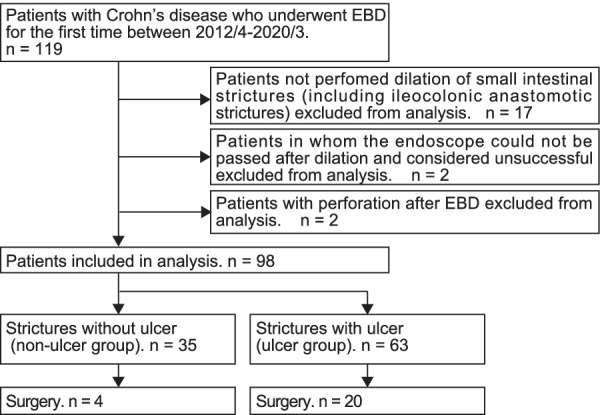
Flow diagram of patient selection. *EBD* endoscopic balloon dilation

Using an electronic medical record system, we identified 119 patients diagnosed with CD who underwent EBD for the first time for an intestinal stricture at Tokyo Medical and Dental University Hospital in Tokyo, Japan, between January 2012 and March 2020. We included patients who underwent initial dilation for small intestinal strictures unable to be passed by the endoscope, and excluded patients treated for ileocolonic anastomotic strictures (n = 17). EBD was considered effective when the endoscope could be successfully passed after the procedure. Unsuccessful EBD was noted in two patients, thereby excluded. Two cases of perforation after EBD were also excluded (n = 2). Finally, 98 patients were included for analysis (Fig. 1). The Ethics Committee of the Tokyo Medical and Dental University approved this study (Approval number. M2020-325). All patient information was anonymized before statistical analysis. We obtained informed consent from all patients (opt-out approach). Furthermore, this study conformed to the Declaration of Helsinki.

### Endoscopic procedure and evaluation

BAE was performed for endoscopic evaluation, diagnosis, and treatment of small intestinal strictures including the terminal ileum in CD patients. We used the single-balloon endoscopy system (SIF-Q260 or SIF-H290S; Olympus, Tokyo, Japan) for BAE. The endoscopic examination was performed as previously reported [21]. Indications for EBD were determined according to the following criteria; stricture that a 9.2 mm-diameter endoscope cannot pass through, stricture length < 5 cm, absence of fistula, absence of deep ulcer, and absence of a steep curvature making dilation difficult [11]. A through-the-scope balloon catheter (CRE Wire-guided Balloon Dilators; Boston Scientific, Marlborough, MA, USA) was used for EBD. The balloon dilator was inserted within the stricture and inflated under direct vision for 2 min.

### Measurements

We identified the date of first EBD for each patient. The most distal stricture that the endoscope could not pass was included for analysis in each patient. Primary outcome was set as the incidence of surgical treatment for the target stricture post-EBD. We measured the duration from the date of EBD to the date of surgery for the stricture, or to the date of the last hospital visit. Therefore, the data of patients who underwent surgery for non–EBD-related strictures were considered censored data. In this study, one patient who was surgically treated for bowel perforation of a site which was not the original targeted stricture was treated as censored data.

The presence of ulcer at the stricture site was noted because we defined mucosal healing as the absence of ulcers at the stricture site in this study. Moreover, we collected the following endoscopic findings as confounders; length of the stricture, whether the stricture was an anastomotic stricture, and the location of stricture (terminal ileum [TI]: ≤ 10 cm from the ileocecal valve, proximal ileum [PI]: 10–300 cm from the ileocecal valve, and jejunum [J]: > 300 cm from the ileocecal valve) [22]. Considering that a small intestinal stricture length of ≥ 2 cm was previously reported as a risk factor for surgery, we categorized stricture lengths into ≥ 2 cm and < 2 cm [12]. The endoscopic disease activity of the stricture was assessed by the presence of ulcer at the stricture, as previously reported [15], and patients were classified accordingly. We did not consider details of EBD (e.g. balloon dilation diameter) as a confounding factor because of its temporality order (i.e. the presence of ulcer is determined before EBD), but they were included in a different model to examine whether the result changed with adjustment. Moreover, a previous report on small intestinal lesions showed that the avoidance rate of intestinal resection increased with dilation diameter of ≥ 15 mm [12], therefore, we categorized balloon dilation diameters into ≥ 15 mm and < 15 mm.

We also collected the following information on potential confounders regarding patients’ characteristics; age at CD diagnosis, sex, smoking status (non-smoker/current smoker/ex-smoker), previous intestinal resection, perianal involvement, and disease location (ileal [L1], ileocolonic [L3]) [23]. The Crohn’s disease activity index (CDAI) score [24], C-reactive protein (CRP), and concomitant treatment at the time of EBD were also noted. The presence of obstructive symptom before EBD was not included as a variable in the analysis because the CDAI score includes abdominal pain. The CDAI score was categorized as ≥ 150 and < 150 [24]. The age at diagnosis was categorized into < 17, 17–40, and > 40 years old [23]. In addition, CRP level was categorized as ≤ 4 mg/L and > 4 mg/L [5].

Since 24 weeks of anti-TNF administration has been reported to be effective for small bowel strictures in Crohn's disease [25], the duration of drug administration in patients who received anti-TNF was also considered as an additional analysis. The duration of anti-TNF administration was categorized as < 24 weeks and ≥ 24 weeks.

To examine whether endoscopic re-dilation was required at follow-up endoscopy, we conducted a subanalysis. Patients who underwent follow-up endoscopy during the observation period were analyzed (n = 71). We examined whether the endoscope reached the target stricture to be analyzed and whether EBD was repeated on the stricture.

### Analysis

Patients’ baseline characteristics were compared according to the presence of ulcer at the stricture site. The difference in survival time according to the presence of ulcer at the stricture site was evaluated by Kaplan–Meier survival analysis. The association between surgery and ulcer at the stricture was examined using a Cox proportional hazards model. First, each explanatory variable was examined by univariate analysis. Then, the variables previously reported as risks for surgery after EBD and those variables with *p* < 0.10 were used for multivariate analysis, which was adjusted for sex, age at diagnosis, and the selected variables. For checking the robustness of the results, sensitivity analysis was conducted, with the balloon dilation diameter included as a variable.

In the subanalysis, we included eligible patients who underwent follow-up BAE. The association between re-dilation and the presence of ulcer at the stricture site was examined using a logistic regression model. Likewise, each explanatory variable underwent univariate analysis. Next, variables with *p* < 0.10 were included in the multivariate analysis, which was adjusted for sex, age at diagnosis, and the selected variables.

All statistical data were analyzed using Stata/MP 16.1 (StataCorp, College Station, TX, USA). Furthermore, *p* < 0.05 was considered statistically significant.

## Results

### Patient demographics

Table 1 summarizes the baseline characteristics of the 98 eligible patients. Ulcer at the stricture site was identified in 63 (64.3%) patients. The proportions of patients with previous intestinal resection, perianal involvement, and CRP > 4 mg/L were significantly higher in the ulcer group than in the non-ulcer group (47.6% vs. 20.0% (*p* = 0.009), 42.9% vs. 20.0% (*p* = 0.027), 28.6% vs. 8.6% (*p* = 0.022), respectively). No significant differences were found in other endoscopic findings (stricture length, anastomotic site stricture, and stricture location). Regarding the results of endoscopic treatment, the proportion of patients undergoing dilation diameter ≥ 15 mm was significantly higher in the non-ulcer group (65.7%) than in the ulcer group (38.1%) (*p* = 0.009). The duration of anti-TNF administration is shown in the Additional file 1: Table S1. No significant correlation was found between the duration of anti-TNF use and the presence of ulcers.

**Table 1:** Baseline characteristics of patients at time of EBD, endoscopic treatment, and outcome (n = 98)

Variables	Total	Presence of ulcer at the stricture		*p* value
		No	Yes	
	98 (100.0%)	35 (35.7%)	63 (64.3%)	
*Baseline characteristics*				
Sex, n (%)				
Men	76 (77.6%)	26 (74.3%)	50 (79.4%)	0.618^†^
Women	22 (22.4%)	9 (25.7%)	13 (20.6%)	
Median age at diagnosis of CD, years (range)	28 (3, 63)	30 (12, 63)	27 (3, 60)	
Age at diagnosis of CD, n (%)				
< 17	10 (10.2%)	4 (11.4%)	6 (9.5%)	1.000^†^
17–40	70 (71.4%)	25 (71.4%)	45 (71.4%)	
> 40	18 (18.4%)	6 (17.1%)	12 (19.1%)	
Median disease duration, years (range)	4.64 (0.00, 32.57)	3.00 (0.00, 31.99)	7.13 (0.00, 32.57)	
Smoking, n (%)				
Never	70 (71.4%)	23 (65.7%)	47 (74.6%)	0.470^†^
Current	16 (16.3%)	8 (22.9%)	8 (12.7%)	
Ex-smoker	12 (12.2%)	4 (11.4%)	8 (12.7%)	
Previous intestinal resection, n (%)				
No	61 (62.2%)	28 (80.0%)	33 (52.4%)	0.009^†^*
Yes	37 (37.8%)	7 (20.0%)	30 (47.6%)	
Perianal involvement, n (%)				
No	64 (65.3%)	28 (80.0%)	36 (57.1%)	0.027^†^*
Yes	34 (34.7%)	7 (20.0%)	27 (42.9%)	
Disease location, n (%)				
L1	54 (55.1%)	22 (62.9%)	32 (50.8%)	0.250^‡^
L3	44 (44.9%)	13 (37.1%)	31 (49.2%)	
Median CDAI score, (range)	78.32 (0.00, 383.52)	63.84 (0.00, 198.97)	84.00 (0.00, 383.52)	
CDAI score, n (%)				
< 150	79 (80.6%)	31 (88.6%)	48 (76.2%)	0.185^†^
≥ 150	19 (19.4%)	4 (11.4%)	15 (23.8%)	
Median CRP, mg/L (range)	1.1 (0.2, 66.4)	1.0 (0.2, 13.2)	1.2 (0.2, 66.4)	
CRP, n (%)				
≤ 4 mg/L	77 (78.6%)	32 (91.4%)	45 (71.4%)	0.022^†^*
> 4 mg/L	21 (21.4%)	3 (8.6%)	18 (28.6%)	
*Endoscopic findings*				
Stricture length, n (%)				
< 2 cm	92 (93.9%)	32 (91.4%)	60 (95.2%)	0.663^†^
≥ 2 cm	6 (6.1%)	3 (8.6%)	3 (4.8%)	
Anastomotic site stricture, n (%)				
No	89 (90.8%)	31 (88.6%)	58 (92.1%)	0.717^†^
Yes	9 (9.2%)	4 (11.4%)	5 (7.9%)	
Location of stricture, n (%)				
TI	21 (21.4%)	8 (22.9%)	13 (20.6%)	0.933^†^
PI	71 (72.4%)	25 (71.4%)	46 (73.0%)	
J	6 (6.1%)	2 (5.7%)	4 (6.3%)	
*Concomitant treatment*				
Corticosteroid, n (%)				
No	87 (88.8%)	31 (88.6%)	56 (88.9%)	1.000^†^
Yes	11 (11.2%)	4 (11.4%)	7 (11.1%)	
5-aminosalicylate, n (%)				
No	35 (35.7%)	14 (40.0%)	21 (33.3%)	0.509^‡^
Yes	63 (64.3%)	21 (60.0%)	42 (66.7%)	
Anti-TNF, n (%)				
No	51 (52.0%)	17 (48.6%)	34 (54.0%)	0.608^‡^
Yes	47 (48.0%)	18 (51.4%)	29 (46.0%)	
Immunomodulator, n (%)				
No	60 (61.2%)	24 (68.6%)	36 (57.1%)	0.266^‡^
Yes	38 (38.8%)	11 (31.4%)	27 (42.9%)	
Anti-TNF + Immunomodulator, n (%)				
No	80 (81.6%)	29 (82.9%)	51 (81.0%)	1.000^†^
Yes	18 (18.4%)	6 (17.1%)	12 (19.0%)	
*Endoscopic treatment*				
Balloon dilation diameter, n (%)				
< 15 mm	51 (52.0%)	12 (34.3%)	39 (61.9%)	0.009^‡^*
≥ 15 mm	47 (48.0%)	23 (65.7%)	24 (38.1%)	
*Outcome*				
Surgery				
No	74 (75.5%)	31 (88.6%)	43 (68.3%)	0.029^†^*
Yes	24 (24.5%)	4 (11.4%)	20 (31.7%)	

*EBD* endoscopic balloon dilation, *CD* Crohn's disease, *CDAI* Crohn's disease activity index, *CRP* C-reactive protein, *TI* terminal ileum, *PI* proximal ileum, *J* jejunum, *TNF* tumor necrosis factor

^†^Fisher's exact test

^‡^Chi-square test

^*^p < 0.05

### Association between ulcer at the stricture and surgical risk

**Fig. 2:**
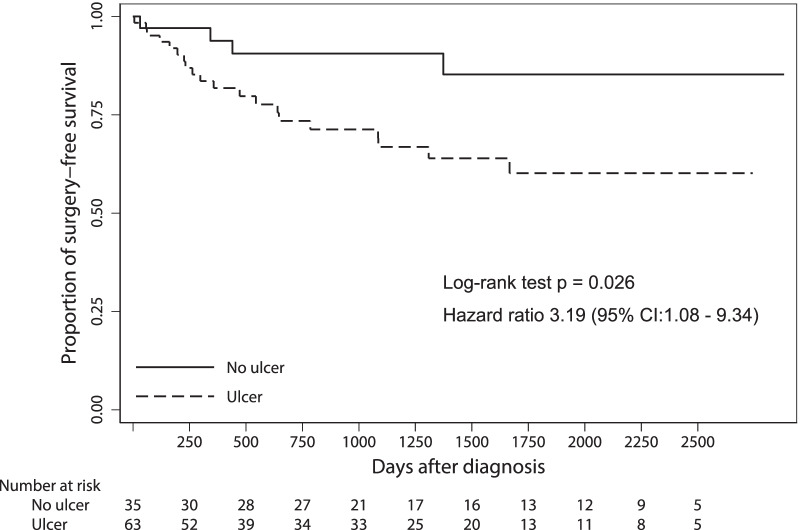
Kaplan–Meier curve showing the cumulative probability of surgery-free survival after the EBD date. *EBD* endoscopic balloon dilation, *CI* confidence interval

A total of 24 patients (24.5%) underwent surgery. The proportion of patients who underwent surgery was significantly higher in the ulcer group than in the non-ulcer group (20 [31.7%] vs. 4 [11.4%] (*p* = 0.029), respectively; Table 1). Figure 2 shows the Kaplan–Meier curve showing the cumulative nonoperative survival rate after the first EBD date. The transition rate to surgery was 19.2% at 1 year and 39.8% at 5 years in the ulcer group. The ulcer group tended to undergo surgery earlier than the non-ulcer group.

Table 2 shows the analysis of Cox proportional hazards models examining the risk of surgery. In the univariate analysis, surgical risk was significantly increased in women (hazard ratio [HR] 2.56; 95% confidence interval [CI] 1.14–5.77; p = 0.023), CDAI score ≥ 150 (HR 2.91; 95% CI 1.23–6.84; *p* = 0.015), and the presence of ulcer at the stricture (HR 3.19; 95% CI 1.08–9.34; *p* = 0.035). Treatment with anti-TNF or combined treatment with anti-TNF and immunomodulators were not associated with the risk of surgery. Other factors showed no significant differences. As previously mentioned, the multivariate analysis was adjusted for sex, age, previous intestinal resection, CDAI score, presence of ulcer at the stricture, and stricture length. However, the ulcer group had a significantly increased risk for surgery even after adjustment for these confounders (HR 4.84; 95% CI 1.58–14.79; *p* = 0.006). Patients with previous intestinal resection had a lower risk for surgery (HR 0.26; 95% CI 0.09–0.76; *p* = 0.014). A stricture length of ≥ 2 cm had a higher surgical risk (HR 3.31; 95% CI 0.68–15.99; *p* = 0.137), but the association was not statistically significant. Factors such as sex, age at diagnosis and CDAI score were not associated with surgical risk in the multivariate analysis.

**Table 2:** Cox proportional hazards model showing the hazard ratios for surgery (n = 98)

Variables at diagnosis		Case of surgery, n	Person-days of follow-up, mean	Univariate			Multivariate		
				HR	95% CI	*p *value	HR	95% CI	*p *value
Sex									
Men	ref	14	1217.974	1.00			1.00		
Women		10	1124.727	2.56	1.14, 5.77	0.023*	1.95	0.74, 5.13	0.176
Age at diagnosis of CD									
< 17		2	1014.3	1.00	0.23, 4.37	0.997	0.55	0.09, 3.31	0.509
17–40	ref	16	1171.543	1.00			1.00		
40 <		6	1397.722	1.29	0.50, 3.30	0.594	1.12	0.42, 2.98	0.816
Smoking									
Never	ref	18	1160.5	1.00					
Current		4	1209	0.95	0.32, 2.82	0.930			
Ex-smoker		2	1394.25	0.55	0.13, 2.39	0.428			
Previous intestinal resection									
No	ref	19	1172.721	1.00			1.00		
Yes		5	1237.135	0.41	0.15, 1.09	0.074	0.26	0.09, 0.76	0.014*
Perianal involvement									
No	ref	15	1336.688	1.00					
Yes		9	934.1765	1.34	0.59, 3.08	0.484			
Disease location									
L1	ref	14	1209.944	1.00					
L3		10	1181.205	0.93	0.41, 2.09	0.853			
CDAI score									
< 150	ref	16	1289.013	1.00			1.00		
≥ 150		8	814.6316	2.91	1.23, 6.84	0.015*	1.83	0.62, 5.41	0.273
CRP									
≤ 4 mg/L	ref	17	1243.896	1.00					
> 4 mg/L		7	1025.238	1.63	0.68, 3.94	0.275			
Endoscopic finding									
Stricture length									
< 2 cm	ref	21	1239.076	1.00			1.00		
≥ 2 cm		3	552.5	3.14	0.91, 10.81	0.069	3.31	0.68, 15.99	0.137
Anastomotic site stricture									
No	ref	22	1189.899	1.00					
Yes		2	1267.667	0.85	0.20, 3.64	0.831			
Location of stricture									
TI	ref	9	1250.667	1.00					
PI		15	1138.563	0.52	0.22, 1.19	0.123			
J		0	1701.333						
Presence of ulcer at the stricture									
No	ref	4	1406.714	1.00			1.00		
Yes		20	1080.556	3.19	1.08, 9.34	0.035*	4.84	1.58, 14.79	0.006*
*Concomitant treatment*									
Corticosteroid									
No	ref	22	1244.345	1.00					
Yes		2	822.9091	0.90	0.21, 3.84	0.883			
5-aminosalicylate									
No	ref	11	1126.629	1.00					
Yes		13	1236.159	0.64	0.29, 1.42	0.271			
Anti-TNF									
No	ref	15	1188.824	1.00					
Yes		9	1205.957	0.62	0.27, 1.41	0.253			
Immunomodulator									
No	ref	15	1218.05	1.00					
Yes		9	1163.868	1.04	0.46, 2.39	0.918			
Anti-TNF + Immunomodulator									
No	ref	20	1190.912	1.00					
Yes		4	1224.278	0.91	0.31, 2.67	0.863			

*ref* reference, *EBD* endoscopic balloon dilation, *CD* Crohn’s disease, *CDAI* Crohn’s disease activity index, *CRP* C-reactive protein, *TI* terminal ileum, *PI* proximal ileum, *J* jejunum, *TNF* tumor necrosis factor, *HR* hazard ratio, *CI* confidence interval. *p < 0.05

Additional file 2: Table S2 shows the analysis of Cox proportional hazards models with adjustment for balloon dilation diameter. Even after adjustment for balloon dilation diameter, the ulcer group had a significantly increased risk for surgery (HR 4.59; 95% CI 1.48–14.24; *p* = 0.008). In both univariate and multivariate analyses, balloon dilation diameter was not associated with surgical risk.

Additional file 3: Table S3 shows the additional analysis of Cox proportional hazards models with adjustment for the duration of anti-TNF administration. In both univariate and multivariate analyses, the duration of anti-TNF administration was not associated with surgical risk.

### Association between ulcer at the stricture and redilation at follow-up BAE

Table 3 summarizes the background of patients included in the secondary endpoint analysis. At follow-up endoscopy, the endoscope reached the stricture to be analyzed in all cases. The median time to follow-up endoscopy was 369 days in the non-ulcer group and 372 days in the ulcer group, with no significant difference. The proportion of patients who underwent redilation at follow-up endoscopy was significantly higher in the ulcer group than in the non-ulcer group (38 [86.4%] vs. 15 [55.6%] (*p* = 0.005), respectively).

**Table 3:** Baseline characteristics at time of EBD, endoscopic treatment, follow-up time, and the outcome of patients undergoing follow-up endoscopy (n = 71)

	Total	Presence of ulcer at the stricture		*p *value
		No	Yes	
Variables	71 (100.0%)	27 (38.0%)	44 (62.0%)	
*Baseline*				
Sex, n (%)				
Men	58 (81.7%)	22 (81.5%)	36 (81.8%)	1.000^†^
Women	13 (18.3%)	5 (18.5%)	8 (18.2%)	
Median age at diagnosis of CD, years (range)	28 (3, 63)	32 (12, 63)	27 (3, 60)	
Age at diagnosis of CD, n (%)				
< 17	8 (11.3%)	4 (14.8%)	4 (9.1%)	0.645^†^
17–40	49 (69.0%)	17 (63.0%)	32 (72.7%)	
> 40	14 (19.7%)	6 (22.2%)	8 (18.2%)	
Median disease duration, years (range)	5.41 (0.00, 32.58)	3.53 (0.12, 31.99)	7.41 (0.00, 32.58)	
Smoking, n (%)				
Never	48 (67.6%)	17 (63.0%)	31 (70.5%)	0.439^†^
Current	13 (18.3%)	7 (25.9%)	6 (13.6%)	
Ex-smoker	10 (14.1%)	3 (11.1%)	7 (15.9%)	
Previous intestinal resection, n (%)				
No	43 (60.6%)	22 (81.5%)	21 (47.7%)	0.006^†^*
Yes	28 (39.4%)	5 (18.5%)	23 (52.3%)	
Perianal involvement, n (%)				
No	48 (67.6%)	22 (81.5%)	26 (59.1%)	0.068^†^
Yes	23 (32.4%)	5 (18.5%)	18 (40.9%)	
Disease location, n (%)				
L1	40 (56.3%)	17 (63.0%)	23 (52.3%)	0.378^‡^
L3	31 (43.7%)	10 (37.0%)	21 (47.7%)	
Median CDAI score, (range)	76.8 (0.00, 269.2)	68.99 (3.94, 198.2)	79.2 (0.00, 269.2)	
CDAI score, n (%)				
< 150	62 (87.3%)	25 (92.6%)	37 (84.1%)	0.467^†^
≥ 150	9 (12.7%)	2 (7.4%)	7 (15.9%)	
Median CRP at time of EBD, mg/L (range)	0.8 (0.2, 66.4)	0.8 (0.2, 13.2)	0.8 (0.2, 66.4)	
CRP at time of EBD, n (%)				
≤ 4 mg/L	56 (78.9%)	25 (92.6%)	31 (70.5%)	0.036^†^*
> 4 mg/L	15 (21.1%)	2 (7.4%)	13 (29.5%)	
*Endoscopic findings*				
Stricture length, n (%)				
< 2 cm	67 (94.4%)	24 (88.9%)	43 (97.7%)	0.151^†^
≥ 2 cm	4 (5.6%)	3 (11.1%)	1 (2.3%)	
Anastomotic site stricture, n (%)				
No	64 (90.1%)	25 (92.6%)	39 (88.6%)	0.701^†^
Yes	7 (9.9%)	2 (7.4%)	5 (11.4%)	
Location of stricture, n (%)				
TI	16 (22.5%)	6 (22.2%)	10 (22.7%)	0.777^†^
PI	50 (70.4%)	20 (74.1%)	30 (68.2%)	
J	5 (7.0%)	1 (3.7%)	4 (9.1%)	
*Concomitant treatment*				
Corticosteroid, n (%)				
No	62 (87.3%)	23 (85.2%)	39 (88.6%)	0.723^†^
Yes	9 (12.7%)	4 (14.8%)	5 (11.4%)	
5-aminosalicylate, n (%)				
No	25 (35.2%)	10 (37.0%)	15 (34.1%)	0.801^‡^
Yes	46 (64.8%)	17 (63.0%)	29 (65.9%)	
Anti-TNF, n (%)				
No	35 (49.3%)	13 (48.1%)	22 (50.0%)	0.880^‡^
Yes	36 (50.7%)	14 (51.9%)	22 (50.0%)	
Immunomodulator, n (%)				
No	45 (63.4%)	18 (66.7%)	27 (61.4%)	0.801^†^
Yes	26 (36.6%)	9 (33.3%)	17 (38.6%)	
Anti-TNF + Immunomodulator, n (%)				
No	59 (83.1%)	23 (85.2%)	36 (81.8%)	1.000^†^
Yes	12 (16.9%)	4 (14.8%)	8 (18.2%)	
*Endoscopic treatment and follow-up time*				
Balloon dilation diameter, n (%)				
< 15 mm	35 (49.3%)	7 (25.9%)	28 (63.6%)	0.003^†^*
≥ 15 mm	36 (50.7%)	20 (74.1%)	16 (36.4%)	
Median time between EBD and follow-up endoscopy, days (range)	371 (89, 1843)	369 (130, 1843)	372 (89, 1163)	0.619^§^
*Outcome*				
Redilation at follow-up endoscopy				
No	18 (25.4%)	12 (44.4%)	6 (13.6%)	0.005^†^*
Yes	53 (74.6%)	15 (55.6%)	38 (86.4%)	

*EBD* endoscopic balloon dilation, *CD* Crohn's disease, *CDAI* Crohn's disease activity index, *CRP* C-reactive protein, *TI* terminal ileum, *PI* proximal ileum, *J* jejunum, *TNF* tumor necrosis factor

^†^Fisher's exact test

^‡^Chi-square test

^§^Wilcoxon rank sum test

^*^p < 0.05

Table 4 shows the results of logistic regression analyses examining the association between redilation and ulcer at the stricture. In the univariate analysis, the risk for redilation was significantly higher in the ulcer group than in the non-ulcer group (odds ratio [OR] 5.07; 95% CI 1.61–15.97; *p* = 0.006). Other factors showed no significant association, and no variable had *p* < 0.10. Even after adjustment for sex and age, the association between ulcer at the stricture and risk of redilation was significantly higher in the ulcer group than in the non-ulcer group (OR 5.69; 95% CI 1.72–18.80; *p* = 0.004).

**Table 4:** Logistic regression model showing the odds ratios for redilation in patients undergoing follow-up endoscopy (n = 71)

Variables at diagnosis		Case of redilation, n	Univariate			Multivariate		
			OR	95% CI	*p *value	OR	95% CI	*p *value
Sex								
Men	ref	44	1.00			1.00		
Women		9	0.72	0.19, 2.69	0.620	0.56	0.13, 2.45	0.445
Age at diagnosis of CD								
< 17		7	2.27	0.25, 20.37	0.464	3.81	0.35, 40.91	0.270
17–40	ref	37	1.00			1.00		
40 <		9	0.58	0.16, 2.08	0.407	0.62	0.15, 2.47	0.495
Smoking								
Never	ref	37	1.00					
Current		8	0.48	0.13, 1.75	0.264			
Ex-smoker		8	1.19	0.22, 6.44	0.841			
Previous intestinal resection								
No	ref	32	1.00					
Yes		21	1.03	0.34, 3.09	0.956			
Perianal involvement								
No	ref	35	1.00					
Yes		18	1.34	0.41, 4.34	0.629			
Disease location								
L1	ref	28	1.00					
L3		25	1.79	0.58, 5.47	0.310			
CDAI score								
< 150	ref	47	1.00					
≥ 150		6	0.64	0.14, 2.87	0.558			
CRP at time of EBD								
≤ 4 mg/L	ref	42	1.00					
> 4 mg/L		11	0.92	0.25, 3.34	0.895			
*Endoscopic findings*								
Stricture length								
< 2 cm	ref	51	1.00					
≥ 2 cm		2	0.31	0.04, 2.41	0.265			
Anastomotic site stricture								
No	ref	48	1.00					
Yes		5	0.83	0.15, 4.72	0.837			
Location of stricture								
TI	ref	14	1.00					
PI		35	0.33	0.07, 1.65	0.178			
J		4	0.57	0.04, 8.05	0.678			
Presence of ulcer at the stricture								
No	ref	15	1.00			1.00		
Yes		38	5.07	1.61, 15.97	0.006*	5.69	1.72, 18.80	0.004*
*Concomitant treatment*								
Corticosteroid								
No	ref	47	1.00					
Yes		6	0.64	0.14, 2.87	0.558			
5-aminosalicylate								
No	ref	19	1.00					
Yes		34	0.89	0.29, 2.77	0.847			
Anti-TNF								
No	ref	24	1.00					
Yes		29	1.90	0.64, 5.65	0.249			
Immunomodulator								
No	ref	31	1.00					
Yes		22	2.48	0.72, 8.57	0.150			
Anti-TNF + Immunomodulator								
No	ref	42	1.00					
Yes		11	4.45	0.53, 37.2	0.168			

*ref* reference, *EBD* endoscopic balloon dilation, *CD* Crohn’s disease, *CDAI* Crohn’s disease activity index, *CRP* C-reactive protein, *TI* terminal ileum, *PI* proximal ileum, *J* jejunum, *TNF* tumor necrosis factor, *OR* odds ratio, *CI* confidence interval. *p < 0.05

Additional file 4: Table S4 shows the results of logistic regression analyses, with adjustment for balloon dilation diameter and time from initial EBD to second endoscopy. When these two variables were added in the multivariate analysis, the risk for redilation was significantly higher in the ulcer group than in the non-ulcer group (OR 4.23; 95% CI 1.18–15.15; *p* = 0.027). Balloon dilation diameter ≥ 15 mm had a significantly lower risk for redilation in the univariate analysis (OR 0.20; 95% CI 0.06–0.70; *p* = 0.012), but the association became nonsignificant in the multivariate analysis (OR 0.30; 95% CI 0.07–1.20; *p* = 0.088). The time from initial EBD to second endoscopy was not associated with risk for redilation in either the univariate or multivariate analysis.

## Discussion

In this study with 98 patients, we found that small intestinal stricture with ulcer had a higher risk for surgery after EBD than small intestinal stricture without ulcer. In addition, small intestinal stricture with ulcer had a higher risk of requiring redilation during follow-up endoscopy. To our knowledge, this study is the first to report the relationship between endoscopic activity at the stricture site and outcome after EBD. Our results suggest that prior mucosal healing of the stricture site may improve the prognosis of EBD for small intestinal strictures in CD.

It is important to evaluate and manage small intestinal strictures in order to avoid surgery, considering that strictures constitute half of the reasons for surgery in CD [26] and multiple intestinal resections for small intestinal strictures are associated with the risk for short bowel syndrome [27]. Although EBD has been reported to be effective for small intestinal strictures [5–12], further improvement in the prognosis post-EBD is required. Mucosal healing is an important therapeutic target in inflammatory bowel disease [28], and it is known to improve the prognosis [18, 19], even when limited to small intestinal lesions [20]. However, previous reports did not take into account whether EBD has been performed for strictures, and the relationship between endoscopic activity and the outcome of strictures after EBD was unclear. The present study clearly showed that ulcerated strictures were associated with surgical risk and that mucosal healing of these strictures could improve the prognosis of patients who undergo EBD. Furthermore, redilation risk was lower when there was absence of ulcer of the stricture compared to when the stricture was endoscopically active, suggesting the frequency of small intestinal endoscopy can possibly be reduced by aiming for mucosal healing before EBD, contributing to reducing patients’ burden.

The guidelines state that the presence of an ulcer is not a contraindication to EBD for CD stricture [16], but if the stricture is accompanied by a deep ulcer, EBD should be avoided [29]. However, the effect of endoscopic activity and EBD, including the presence of ulcer, was unexamined so far. In this study, we clarified the prognosis after EBD for small intestinal stricture with ulcer. In previous reports, the rate of transition to surgery after EBD, without considering the endoscopic activity of the stricture site, was 30.1% and 42.9% at 12 and 24 months after EBD, respectively [14]. Another study reported that the transition rates to surgery at 1 year and 5 years after EBD were 26.0% and 45.6%, respectively [12]. In the present study, the transition rate to surgery after EBD was slightly lower than the rates obtained by the previous studies. The possible reasons were that the present analysis was limited to initial EBD and that the indication was determined by whether the endoscope was able to pass the stricture, not by the presence of clinical symptoms.

A previous study reported stricture length did not affect the likelihood of the patient undergoing surgery after EBD [12]. According to our multivariate analysis, a stricture length of ≥ 2 cm had a higher surgical risk, but the association was not statistically significant (Table 2). These results are consistent with previously reported results.

The balloon dilation diameter was significantly lower in the ulcer group (*p* = 0.009, Table 1). The presence of ulcer may have led to the selection of a smaller diastolic balloon diameter during endoscopy. Considering the influence of balloon dilation diameter, we performed multivariate analysis (Additional file 2: Table S2, Additional file 4: Table S4), with balloon dilation diameter as a covariate, and found that the diastolic diameter was not associated with the risk for surgery or redilation.

Previous two trials [25, 30] indicated that anti-TNF treatment is effective in alleviating stenosis. In the present study, neither anti-TNF treatment nor combination (anti-TNF + immunomodulator) treatment was associated with surgical risk (Table 2). Duration of anti-TNF treatment also showed no association with surgical risk (Additional file 3: Table S3). Possible reasons for the ineffectiveness of anti-TNF therapy in this study is that this study excluded strictures with deep ulcers, which are good indications for anti-inflammatory therapy, and that this study included patients with low therapeutic response who had residual strictures requiring EBD after starting anti-TNF therapy.

The present study has several limitations. Firstly, it is a single-center, retrospective, observational study. Although the small number of sample from one university hospital might limit the generalizability of the findings, we found that the present study’s rate of surgical outcome for EBD did not differ greatly from previous reports [12, 14]. Secondly, we did not consider change in medical treatment after the initial EBD. Though treatment change may influence the prognosis after EBD, this bias was thought not to be severe because one previous study reported that intensification of treatment at the onset of abdominal pain before EBD did not affect the prognosis after EBD [12]. However, no previous reports have investigated for treatment intensification based on the findings of EBD. This may affect the prognosis, and future prospective studies are desirable. Thirdly, the strictures were evaluated only for the most distal lesion, and multiple strictures were not considered. We did not include the number of strictures as a factor because this study focused on the endoscopic evaluation of mucosal activity (presence of ulcer at the target stricture). However, in a previous report, the frequency of requiring redilation after EBD was not significantly different between patients with a single stricture and multiple strictures [31]. Another report showed that surgical risk after EBD was not significantly different between patients with a single stricture and multiple strictures (OR 0.548; 95% CI 0.207–1.429) [12]. An analysis of prognoses after EBD of CD strictures including colorectal strictures showed that the presence of multiple strictures was not associated with increased surgical risk (HR 1.6; 95% CI 0.9–2.8) [32]. Thus, bias caused by multiple strictures on our results might be minimal.

In conclusion, small intestinal stricture in CD patients with ulcer is associated with a higher risk for surgery or redilation after EBD than small intestinal stricture without ulcer. Achievement of mucosal healing before EBD may improve the prognosis of small intestinal stricture in CD.

## Supplementary Information

**Additional file 1: Table S1.** Baseline characteristics of the duration of TNF use in patients at the time of EBD (n = 98).

**Additional file 2: Table S2.** Cox proportional hazards model showing the hazard ratios for surgery (n = 98), adding balloon dilation diameter as a variable.

**Additional file 3: Table S3.** Cox proportional hazards model showing the hazard ratios for surgery; adding duration of anti-TNF use as a variable.

**Additional file 4: Table S4.** Logistic regression model showing the odds ratios for redilation of patients who underwent follow-up endoscopy (n = 71), adding endoscopic treatment and follow-up time as variables.

## Data Availability

The datasets used and/or analysed during the current study are available from the corresponding author on reasonable request.

## Abbreviations

CD: Crohn’s disease
EBD: Endoscopic balloon dilation
BAE: Balloon-assisted enteroscopy
TI: Terminal ileum
PI: Proximal ileum
J: Jejunum
CDAI: Crohn’s disease activity index
HR: Hazard ratio
CI: Confidence interval
OR: Odds ratio
