# Supplementary material for: Mucosal healing of small intestinal stricture is associated with improved prognosis post-dilation in Crohn’s disease
Source: BMC Gastroenterol. 2022 May 4;22:218. doi: 10.1186/s12876-022-02300-2 (PMC9066722; doi:10.1186/s12876-022-02300-2)
Supplement: Supplementary file 1 — Additional file 1: Table S1. Baseline characteristics of the duration of TNF use in patients at the time of EBD (n = 98). [file 12876_2022_2300_MOESM1_ESM.docx]

**Supplementary Table 1.** Baseline characteristics of the duration of TNF use in patients at the time of EBD (n = 98).

|  |  | Presence of ulcer at the stricture | |  |  |
| --- | --- | --- | --- | --- | --- |
|  | Total | No | Yes |  |  |
| Variables | 98 (100.0%) | 35 (35.7%) | 63 (64.3%) | P-value | |
| **Concomitant treatment: Anti-TNF use** |  |  |  |  |  |
| No anti-TNF use, n (%) | 51 (52.0%) | 17 (48.6%) | 34 (54.0%) | 0.172 | † |
| Anti-TNF use <24 weeks, n (%) | 5 (5.1%) | 0 (0.0%) | 5 (7.9%) |  |  |
| Anti-TNF use ≥24 weeks, n (%) | 42 (42.9%) | 18 (51.4%) | 24 (38.1%) |  |  |
|  |  |  |  |  |  |
| Median duration of anti-TNF therapy   in the Anti-TNF use group, years (range) | 3.64 (0.08, 13.66) | 3.28 (0.77, 11.44) | 3.86 (0.08, 13.66) | 0.694 | ‡ |
|  |  |  |  |  |  |
| Abbreviations: EBD, endoscopic balloon dilation; TNF, tumor necrosis factor. | | | | | |
| †Fisher's exact test, ‡Wilcoxon rank sum test |  |  |  |  |  |
