# Supplementary material for: Mucosal healing of small intestinal stricture is associated with improved prognosis post-dilation in Crohn’s disease
Source: BMC Gastroenterol. 2022 May 4;22:218. doi: 10.1186/s12876-022-02300-2 (PMC9066722; doi:10.1186/s12876-022-02300-2)
Supplement: Supplementary file 3 — Additional file 3: Table S3. Cox proportional hazards model showing the hazard ratios for surgery; adding duration of anti-TNF use as a variable. [file 12876_2022_2300_MOESM3_ESM.docx]

**Supplementary Table 3.** Cox proportional hazards model showing the hazard ratios for surgery; adding duration of anti-TNF use as a variable.

|  |  |  |  |  |  | Univariate | | |  | Multivariate | | |
| --- | --- | --- | --- | --- | --- | --- | --- | --- | --- | --- | --- | --- |
| Variables at diagnosis |  |  | case of surgery, n | Person-days of follow-up, mean |  | HR | 95% CI | P-value |  | HR | 95% CI | P-value |
| Sex | Men | ref | 14 | 1217.974 |  | 1.00 |  |  |  | 1.00 |  |  |
|  | Women |  | 10 | 1124.727 |  | 2.56 | 1.14, 5.77 | 0.023* |  | 1.92 | 0.72, 5.09 | 0.190 |
|  |  |  |  |  |  |  |  |  |  |  |  |  |
| Age at diagnosis of CD | <17 |  | 2 | 1014.3 |  | 1.00 | 0.23, 4.37 | 0.997 |  | 0.54 | 0.09, 3.34 | 0.504 |
|  | 17-40 | ref | 16 | 1171.543 |  | 1.00 |  |  |  | 1.00 |  |  |
|  | 40< |  | 6 | 1397.722 |  | 1.29 | 0.50, 3.30 | 0.594 |  | 1.10 | 0.38, 3.19 | 0.856 |
|  |  |  |  |  |  |  |  |  |  |  |  |  |
| Smoking | Never | ref | 18 | 1160.5 |  | 1.00 |  |  |  |  |  |  |
|  | Current |  | 4 | 1209 |  | 0.95 | 0.32, 2.82 | 0.930 |  |  |  |  |
|  | Ex-smoker | | 2 | 1394.25 |  | 0.55 | 0.13, 2.39 | 0.428 |  |  |  |  |
|  |  |  |  |  |  |  |  |  |  |  |  |  |
| Previous intestinal resection | No | ref | 19 | 1172.721 |  | 1.00 |  |  |  | 1.00 |  |  |
|  | Yes |  | 5 | 1237.135 |  | 0.41 | 0.15, 1.09 | 0.074 |  | 0.27 | 0.09, 0.82 | 0.021* |
|  |  |  |  |  |  |  |  |  |  |  |  |  |
| Perianal involvement | No | ref | 15 | 1336.688 |  | 1.00 |  |  |  |  |  |  |
|  | Yes |  | 9 | 934.1765 |  | 1.34 | 0.59, 3.08 | 0.484 |  |  |  |  |
|  |  |  |  |  |  |  |  |  |  |  |  |  |
| Disease location | L1 | ref | 14 | 1209.944 |  | 1.00 |  |  |  |  |  |  |
|  | L3 |  | 10 | 1181.205 |  | 0.93 | 0.41, 2.09 | 0.853 |  |  |  |  |
|  |  |  |  |  |  |  |  |  |  |  |  |  |
| CDAI score | <150 | ref | 16 | 1289.013 |  | 1.00 |  |  |  | 1.00 |  |  |
|  | ≥150 |  | 8 | 814.6316 |  | 2.91 | 1.23, 6.84 | 0.015* |  | 1.85 | 0.62, 5.48 | 0.267 |
|  |  |  |  |  |  |  |  |  |  |  |  |  |
| CRP | ≤4 mg/L | ref | 17 | 1243.896 |  | 1.00 |  |  |  |  |  |  |
|  | >4 mg/L |  | 7 | 1025.238 |  | 1.63 | 0.68, 3.94 | 0.275 |  |  |  |  |
|  |  |  |  |  |  |  |  |  |  |  |  |  |
| Endoscopic findings |  |  |  |  |  |  |  |  |  |  |  |  |
| Stricture length | <2 cm | ref | 21 | 1239.076 |  | 1.00 |  |  |  | 1.00 |  |  |
|  | ≥2 cm |  | 3 | 552.5 |  | 3.14 | 0.91, 10.81 | 0.069 |  | 3.41 | 0.70, 16.71 | 0.131 |
|  |  |  |  |  |  |  |  |  |  |  |  |  |
| Anastomotic site stricture | No | ref | 22 | 1189.899 |  | 1.00 |  |  |  |  |  |  |
|  | Yes |  | 2 | 1267.667 |  | 0.85 | 0.20, 3.64 | 0.831 |  |  |  |  |
|  |  |  |  |  |  |  |  |  |  |  |  |  |
| Location of stricture | TI | ref | 9 | 1250.667 |  | 1.00 |  |  |  |  |  |  |
|  | PI |  | 15 | 1138.563 |  | 0.52 | 0.22, 1.19 | 0.123 |  |  |  |  |
|  | J |  | 0 | 1701.333 |  |  |  |  |  |  |  |  |
|  |  |  |  |  |  |  |  |  |  |  |  |  |
| Presence of ulcer at the stricture | No | ref | 4 | 1406.714 |  | 1.00 |  |  |  | 1.00 |  |  |
|  | Yes |  | 20 | 1080.556 |  | 3.19 | 1.08, 9.34 | 0.035* |  | 4.57 | 1.43, 14.61 | 0.010* |
|  |  |  |  |  |  |  |  |  |  |  |  |  |
| Concomitant treatment: |  |  |  |  |  |  |  |  |  |  |  |  |
| Corticosteroid | No | ref | 22 | 1244.345 |  | 1.00 |  |  |  |  |  |  |
|  | Yes |  | 2 | 822.9091 |  | 0.90 | 0.21, 3.84 | 0.883 |  |  |  |  |
|  |  |  |  |  |  |  |  |  |  |  |  |  |
| 5-aminosalicylate | No | ref | 11 | 1126.629 |  | 1.00 |  |  |  |  |  |  |
|  | Yes |  | 13 | 1236.159 |  | 0.64 | 0.29, 1.42 | 0.271 |  |  |  |  |
|  |  |  |  |  |  |  |  |  |  |  |  |  |
| Immunomodulator | No | ref | 15 | 1218.05 |  | 1.00 |  |  |  |  |  |  |
|  | Yes |  | 9 | 1163.868 |  | 1.04 | 0.46, 2.39 | 0.918 |  |  |  |  |
|  |  |  |  |  |  |  |  |  |  |  |  |  |
| Concomitant treatment: Anti-TNF use |  |  |  |  |  |  |  |  |  |  |  |  |
| No anti-TNF use, n (%) |  | ref | 15 | 1188.824 |  | 1.00 |  |  |  | 1.00 |  |  |
| Anti-TNF use <6 months, n (%) |  |  | 2 | 1356.4 |  | 1.09 | 0.25, 4.80 | 0.904 |  | 1.20 | 0.25, 5.79 | 0.824 |
| Anti-TNF use ≥6 months, n (%) |  |  | 7 | 1188.048 |  | 0.55 | 0.22, 1.35 | 0.191 |  | 0.88 | 0.31, 2.53 | 0.815 |
|  |  |  |  |  |  |  |  |  |  |  |  |  |
| Abbreviations: ref, reference; EBD, endoscopic balloon dilation; CD, Crohn's disease; CDAI, Crohn's disease activity index; CRP, C-reactive protein;  TI, terminal ileum; PI, proximal ileum; J, jejunum; TNF, tumor necrosis factor; HR, hazard ratio; CI, confidence interval. | | | | | | | | | | | | |
|  |  |  |  |  |  |  |  |  |  |  |  |  |
| *p<0.05 |  |  |  |  |  |  |  |  |  |  |  |  |
