# Supplementary material for: Mucosal healing of small intestinal stricture is associated with improved prognosis post-dilation in Crohn’s disease
Source: BMC Gastroenterol. 2022 May 4;22:218. doi: 10.1186/s12876-022-02300-2 (PMC9066722; doi:10.1186/s12876-022-02300-2)
Supplement: Supplementary file 4 — Additional file 4: Table S4. Logistic regression model showing the odds ratios for redilation of patients who underwent follow-up endoscopy (n = 71), adding endoscopic treatment and follow-up time as variables. [file 12876_2022_2300_MOESM4_ESM.docx]

**Supplementary Table 4.** Logistic regression model showing the odds ratios for redilation of patients who underwent follow-up endoscopy (n = 71), adding endoscopic treatment and follow-up time as variables.

|  |  |  |  |  | Univariate | | |  | Multivariate | | |
| --- | --- | --- | --- | --- | --- | --- | --- | --- | --- | --- | --- |
| Variables at diagnosis |  |  | case of redilation, n |  | OR | 95% CI | P-value |  | OR | 95% CI | P-value |
| Sex | Men | ref | 44 |  | 1.00 |  |  |  | 1.00 |  |  |
|  | Women |  | 9 |  | 0.72 | 0.19, 2.69 | 0.620 |  | 0.78 | 0.17, 3.69 | 0.757 |
|  |  |  |  |  |  |  |  |  |  |  |  |
| Age at diagnosis of CD | <17 |  | 7 |  | 2.27 | 0.25, 20.37 | 0.464 |  | 3.50 | 0.30, 41.31 | 0.321 |
|  | 17-40 | ref | 37 |  | 1.00 |  |  |  | 1.00 |  |  |
|  | 40< |  | 9 |  | 0.58 | 0.16, 2.08 | 0.407 |  | 0.76 | 0.17, 3.33 | 0.713 |
|  |  |  |  |  |  |  |  |  |  |  |  |
| Smoking | Never | ref | 37 |  | 1.00 |  |  |  |  |  |  |
|  | Current |  | 8 |  | 0.48 | 0.13, 1.75 | 0.264 |  |  |  |  |
|  | Ex-smoker | | 8 |  | 1.19 | 0.22, 6.44 | 0.841 |  |  |  |  |
|  |  |  |  |  |  |  |  |  |  |  |  |
| Previous intestinal resection | No | ref | 32 |  | 1.00 |  |  |  |  |  |  |
|  | Yes |  | 21 |  | 1.03 | 0.34, 3.09 | 0.956 |  |  |  |  |
|  |  |  |  |  |  |  |  |  |  |  |  |
| Perianal involvement | No | ref | 35 |  | 1.00 |  |  |  |  |  |  |
|  | Yes |  | 18 |  | 1.34 | 0.41, 4.34 | 0.629 |  |  |  |  |
|  |  |  |  |  |  |  |  |  |  |  |  |
| Disease location | L1 | ref | 28 |  | 1.00 |  |  |  |  |  |  |
|  | L3 |  | 25 |  | 1.79 | 0.58, 5.47 | 0.310 |  |  |  |  |
|  |  |  |  |  |  |  |  |  |  |  |  |
| CDAI score | <150 | ref | 47 |  | 1.00 |  |  |  |  |  |  |
|  | ≥150 |  | 6 |  | 0.64 | 0.14, 2.87 | 0.558 |  |  |  |  |
|  |  |  |  |  |  |  |  |  |  |  |  |
| CRP at time of EBD | ≤4 mg/L | ref | 42 |  | 1.00 |  |  |  |  |  |  |
|  | >4 mg/L |  | 11 |  | 0.92 | 0.25, 3.34 | 0.895 |  |  |  |  |
|  |  |  |  |  |  |  |  |  |  |  |  |
| Endoscopic findings |  |  |  |  |  |  |  |  |  |  |  |
| Stricture length | <2 cm | ref | 51 |  | 1.00 |  |  |  |  |  |  |
|  | ≥2 cm |  | 2 |  | 0.31 | 0.04, 2.41 | 0.265 |  |  |  |  |
|  |  |  |  |  |  |  |  |  |  |  |  |
| Anastomotic site stricture | No | ref | 48 |  | 1.00 |  |  |  |  |  |  |
|  | Yes |  | 5 |  | 0.83 | 0.15, 4.72 | 0.837 |  |  |  |  |
|  |  |  |  |  |  |  |  |  |  |  |  |
| Location of stricture | TI | ref | 14 |  | 1.00 |  |  |  |  |  |  |
|  | PI |  | 35 |  | 0.33 | 0.07, 1.65 | 0.178 |  |  |  |  |
|  | J |  | 4 |  | 0.57 | 0.04, 8.05 | 0.678 |  |  |  |  |
|  |  |  |  |  |  |  |  |  |  |  |  |
| Presence of ulcer on the stricture | No | ref | 15 |  | 1.00 |  |  |  | 1.00 |  |  |
|  | Yes |  | 38 |  | 5.07 | 1.61, 15.97 | 0.006* |  | 4.23 | 1.18, 15.15 | 0.027* |
|  |  |  |  |  |  |  |  |  |  |  |  |
| Concomitant treatment |  |  |  |  |  |  |  |  |  |  |  |
| Corticosteroid | No | ref | 47 |  | 1.00 |  |  |  |  |  |  |
|  | Yes |  | 6 |  | 0.64 | 0.14, 2.87 | 0.558 |  |  |  |  |
|  |  |  |  |  |  |  |  |  |  |  |  |
| 5-aminosalicylate | No | ref | 19 |  | 1.00 |  |  |  |  |  |  |
|  | Yes |  | 34 |  | 0.89 | 0.29, 2.77 | 0.847 |  |  |  |  |
|  |  |  |  |  |  |  |  |  |  |  |  |
| anti-TNF | No | ref | 24 |  | 1.00 |  |  |  |  |  |  |
|  | Yes |  | 29 |  | 1.90 | 0.64, 5.65 | 0.249 |  |  |  |  |
|  |  |  |  |  |  |  |  |  |  |  |  |
| Immunomodulator | No | ref | 31 |  | 1.00 |  |  |  |  |  |  |
|  | Yes |  | 22 |  | 2.48 | 0.72, 8.57 | 0.150 |  |  |  |  |
|  |  |  |  |  |  |  |  |  |  |  |  |
| Anti-TNF + Immunomodulator | No | ref | 42 |  | 1.00 |  |  |  |  |  |  |
|  | Yes |  | 11 |  | 4.45 | 0.53, 37.2 | 0.168 |  |  |  |  |
| Endoscopic treatment and follow-up |  |  |  |  |  |  |  |  |  |  |  |
| Balloon dilation diameter | <15mm | ref | 31 |  | 1.00 |  |  |  |  |  |  |
|  | ≥15mm |  | 22 |  | 0.20 | 0.06, 0.70 | 0.012* |  | 0.30 | 0.07, 1.20 | 0.088 |
|  |  |  |  |  |  |  |  |  |  |  |  |
| Time from initial EBD to follow-up endoscopy |  |  | 53 |  | 1.00 | 1.00, 1.00 | 0.806 |  | 1.00 | 1.00, 1.00 | 0.432 |

Abbreviations: ref, reference; EBD, endoscopic balloon dilation; CD, Crohn's disease; CDAI, Crohn's disease activity index; CRP, C-reactive protein;

TI, terminal ileum; PI, proximal ileum; J, jejunum; TNF, tumor necrosis factor; OR, odds ratio; CI, confidence interval. *p<0.05
